# An Environmental and Economic Evaluation of Pyrolysis for Energy Generation in Taiwan with Endogenous Land Greenhouse Gases Emissions

**DOI:** 10.3390/ijerph110302973

**Published:** 2014-03-11

**Authors:** Chih-Chun Kung, Bruce A. McCarl, Chi-Chung Chen

**Affiliations:** 1 Institute of Poyang Lake Eco-economics, Jiangxi University of Finance and Economics, Nanchang 330013, China; 2 Department of Agricultural Economics, Texas A&M University, College Station, TX 77843, USA; E-Mail: mccarl@tamu.edu; 3 Department of Applied Economics, National Chung-Hsing University, Taichung 404, Taiwan;E-Mail: mayjune@nchu.edu.tw

**Keywords:** pyrolysis, bioenergy, biochar, land GHG emissions

## Abstract

Taiwan suffers from energy insecurity and the threat of potential damage from global climate changes. Finding ways to alleviate these forces is the key to Taiwan’s future social and economic development. This study examines the economic and environmental impacts when ethanol, conventional electricity and pyrolysis-based electricity are available alternatives. Biochar, as one of the most important by-product from pyrolysis, has the potential to provide significant environmental benefits. Therefore, alternative uses of biochar are also examined in this study. In addition, because planting energy crops would change the current land use pattern, resulting in significant land greenhouse gases (GHG) emissions, this important factor is also incorporated. Results show that bioenergy production can satisfy part of Taiwan’s energy demand, but net GHG emissions offset declines if ethanol is chosen. Moreover, at high GHG price conventional electricity and ethanol will be driven out and pyrolysis will be a dominant technology. Fast pyrolysis dominates when ethanol and GHG prices are low, but slow pyrolysis is dominant at high GHG price, especially when land GHG emissions are endogenously incorporated. The results indicate that when land GHG emission is incorporated, up to 3.8 billion kWh electricity can be produced from fast pyrolysis, while up to 2.2 million tons of CO_2_ equivalent can be offset if slow pyrolysis is applied.

## 1. Introduction

Taiwan is heavily reliant on imported fossil fuels. To enhance energy security, there is interest in domestic energy production. Climate change is also a concern for Taiwan with defenseless warming, sea level rises and increased incidence of tropical cyclones [1]. Energy based CO_2_ emissions could be a driving force for these disasters [1]. Collectively these forces have raised interest in examining low emission, domestic energy sources. Renewable energy produced from agricultural feedstocks (hereafter called bioenergy) is one such possibility. 

Bioenergy production requires substantial use of land resources, another scarce resource in Taiwan. However, after joining the World Trade Organization (WTO), 280,000 hectares of Taiwan’s agricultural land were idled due to reductions in subsidies and increases in imports. This provides some land that could be used for bioenergy feedstock production. 

Although bioenergy has the potential to enhance Taiwan’s energy security and reduce its GHG emissions [2], one important factor that may affect the net benefits is the total set of land and other based GHG emissions involved with feedstock production. When agricultural land is converted to other uses, N_2_O emissions will change and offset CO_2_ energy related emission reductions. If the change is small this can be neglected. Unfortunately, this change can be large [3,4]. For this reason, endogenous incorporation of land GHG emissions could have a significant impact on bioenergy production and emissions reduction. 

Therefore, examining the desirability of bioenergy production without considering all GHG emissions may lead to an incorrect conclusion. This study examines the economic and environmental performance of a set of bioenergy production strategies including production of ethanol, direct firing of electricity production and pyrolysis-based electricity. They will be evaluated under a range of energy and GHG prices. Performance will be considered in terms of GHG emissions, energy production and economic implications. The work will simultaneously consider multiple bioenergy technologies, multiple energy crops, multiple energy and GHG prices, alternative uses of products from pyrolysis and CO_2_ emissions from land use change.

## 2. Literature Review

Taiwan can produce bioenergy in the forms of bioethanol, direct feedstock combustion biopower (conventional electricity) and biopower using products produced through pyrolysis (pyrolysis-based electricity). Since these three technologies are not mutually exclusive and can be employed at the same time, the study considers all combinations. 

Pyrolysis involves heating biomass in the absence of oxygen and results in the decomposition of the biomass into biooil, biogas and biochar, all of which can be used to generate electricity. Depending on the heating rate and time staying in the machine, pyrolysis could be categorized as fast pyrolysis and slow pyrolysis. The main difference between fast and slow pyrolysis is that fast pyrolysis yields more biooil, while slow pyrolysis yields more biochar [5,6]. Biochar can be used as an energy source or as a soil amendment [7,8,9,10,11]. As a soil amendment biochar increases soil water and nutrient holding capacity plus seed germination rates and crop yields. In terms of water holding capacity, Glaser *et al.* [12] find that soil water retention increased by 18% after biochar application. In terms of nutrient savings, the application of biochar has been found to increase the efficiency of nutrients as discussed in Steiner *et al.* [13]. Lehmann *et al.* [14] also indicates that biochar application would lead to a reduction of N leaching by 60 percent with an accompanying 20% savings in fertilizer need. On seed germination several studies find that biochar improves seed germination rate [15,16]. In terms of crop yield enhancement, Lehmann [8] finds that biochar increases the plants available nutrients and in turn crop yields. Crop yield increases have also been found by [13,17,18,19,20] with yield increases ranged from 44% to 249%. Nehls [21] finds rice yield increases ranging from 115% to 320%. Biochar is also stable in the soil [13] and offers a chance to sequester carbon [8].

Based on these data we assume that rice yields will increase by 5% when biochar is applied and use that the seed and nutrient savings are based on Lehmann *et al.*’s study [13] (20 and 10 %, respectively) while water savings are assumed to be 10%. In addition, since water is usually produced during pyrolysis and reduces the heating value, so it is important to remove water from the liquid content. Since electricity and biochar production vary depending on the pyrolysis systems, we examine fast and slow forms of pyrolysis techniques and alternatives uses of biochar. 

Lifecycle analysis [22] has been used to examine GHG emissions from agriculture and bioenergy production in a number of settings. Schaufler *et al.* [23] showed that changes in land-use strongly affected GHG fluxes from cropland, grassland, forests and wetland. Grover *et al.* [4] pointed out that soil-based GHG emissions increase from 53 to 70 t CO_2_-equivalents after land use change. They found that N_2_O and CO_2_ emissions were highest from grassland soils. Baldos [24] found that the direct lifecycle GHG emissions of corn ethanol fuel can exceed the 20% GHG reduction requirement in the USA renewable fuel standard. Baggs *et al.* [25] found that zero tillage resulted in higher N_2_O emissions than conventional tillage and N_2_O emissions were generally correlated with CO_2_ emissions. Farquharson and Baldock [26] indicated that adding N fertilizers will increase N_2_O emissions due to nitrification and denitrification process. Wang *et al.*, [27] and Searchinger *et al.* [28] examined the impacts of emissions from global land use changes finding they can substantially offset GHG net emission gains. Other studies have focused on the land use change emissions when specific land types are cultivated for cropland use [29,30,31]. 

## 3. Model Structure

The study will be done using an agricultural sector model. The model used herein is based on price endogenous mathematical programming, which is originally illustrated by Samuelson [32] who showed a perfectly competitive equilibrium can be simulated by solving an optimization model that maximizes the consumers’ plus producers’ surplus. In particular we will use Chen and Chang’s [33] Taiwan Agricultural Sector Model (TASM) that we extend TASM to cover bioenergy crop production. 

The TASM is a multi-product partial equilibrium model based on the previous work [34,35,36,37]. TASM has been used in many policy-related studies such as Chang [36] and Chen and Chang [33]. The current version covers production in 15 subregions aggregated into four major market regions. It incorporates price-dependent product demand for 60 traditional crops, five floral crops, seven livestock species, three types of forests (conifers, hardwoods, and bamboo), and 27 secondary commodities. The total value of these primary commodities accounts for more than 85 percent of Taiwan’s total agricultural product value. Availability of cropland, pasture land, set aside and forest land plus crop and livestock mix constraints are specified at the sub-regional level. Input markets for farm labor are specified at the regional level with supply curves. 

### 3.1. Modified Taiwan Agricultural Sector Model

For this analysis we extend the TASM version of Chen and Chang [33] adding features related to farm support, bioenergy and GHG emissions. The algebraic form of the modified TASM is as follows:

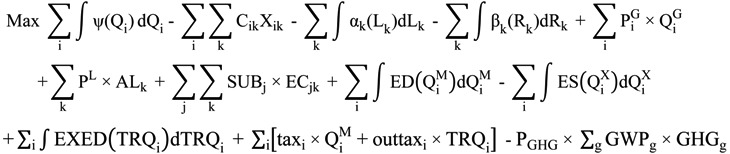
(1)
subject to:

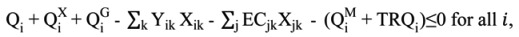
(2)

Σ_i_X_ik_ + AL_k_ + Σ_j_EC_jk_ - L_k_≤0 for all *k*, (3)

Σ_i_f_ik_X_ik_ - Σ_j_f_jk_X_jk_ - R_k_≤0 for all *k*, (4)

Σ_i,k_E_gik_X_ik_-Baseline_g_ = GHG_g_ for all *g* (5)

Equation (1) is the objective function. The area under the domestic demand curve is the 1st term while input costs are in the 2nd term. Then the area under the cropland and labor supply curves are in the 3rd and 4th terms, respectively. The 5th, 6th and 7th terms reflect the government subsidies on rice purchase, set-aside lands and the planting of energy crops. The 8th and 9th terms represent the area under the rest of world export excess demand curve and the 10th term stands for the area under the rest of world excess supply curve. The 11th term is tariff revenue. The final term models GHG offset payments under a carbon dioxide equivalent price. 

Equation (2) is the balance constraint for commodities. The first three terms give alternative demands which includes domestic demand (Q_i_), export demand (Q_i_^X^), and government purchases (Q_i_^G^). The last two terms in the supply-demand balance constraint represents the supply side and include domestic production (Σ_k_Y_ik_X_ik_) and imports (Q_i_^M^ + TRQ_i_). Table 1 depicts the details of variables.

**Table 1:** Variables and their descriptions.

Q_i_	Domestic Demand of Product
Q_i_^G^	Government purchases quantity for price supported product
Q_i_^M^	Import quantity of product
Q_i_^X^	Export quantity of product
ψ(Q_i_)	Inverse demand function of product
P_i_^G^	Government purchase price on product
C_ik_	Purchased input cost in region for producing product
X_ik_	Land use for commodities produced in region
X_jk_	Land use for energy crop produced in region
L_k_	Land supply in region
α_k_(L_k_)	Land inverse supply in region
R_k_	Labor supply in region
β_k_(L_k_)	Labor inverse supply in region
P^L^	Set-aside subsidy
AL_k_	Set-aside acreage in region
SUB_j_	Subsidy on planting energy crop
EC_jk_	Planted acreage of energy crop in region
ED(Q_i_^M^)	Inverse excess import demand curve for product
ES(Q_i_^X^)	Inverse excess export supply curve for product
TRQ_i_	Import quantity exceeding the quota for product
EXED(TRQ_i_)	Inverse excess demand curve of product that the import quantity is exceeding quota
tax_i_	Import tariff for product
outtax_i_	Out-of-quota tariff for product
Y_ik_	Per hectare yield of commodity produced in region
E_gik_	greenhouse gas emission from product in region
P_GHG_	Price of GHG gas
GWP_g_	Global warming potential of greenhouse gas
GHG_g_	Net greenhouse gas emissions of gas
Baseline_g_	Greenhouse gas emissions under the baseline of the gas

Equations (3) and (4) are the resource endowment constraints. Equation (3) controls cropland insuring that planted land plus set-aside land cannot exceed total land and reflects competition by agricultural crops, energy crops and set-aside hectares. Equation (4) is the constraint for other resources such as fertilizer, irrigation and labor requirement. Equation (5) is the net greenhouse gas balance which accounts for the net gain in emissions relative to the baseline as in McCarl and Schneider [38]. 

### 3.2. Modeling Farm Support Policy

In order to incorporate ongoing domestic policies that support rice prices and set-aside cropland, the modified TASM required the addition of variables that reflected the government rice purchasing program and the set-aside program. The rice purchasing program provides farmers with a guaranteed price that is higher than the market equilibrium price. Letting *P_i_^G^* be the weighted government guaranteed purchase price and *Q_i_^G^* be the total amount of government purchase. The farm revenue realized from the government rice purchase program is added into the objective function as an additional farm revenue source. At the same time, it removes rice from the market place up to the amount allowed.

The other set of policy variables related to the land set-aside program are discussed next. If farmers choose to participate in this program, then those farmers will receive a set-aside payment (*P^L^*). The purpose of the objective function that includes consumer and producer surplus is to derive the equilibrium under a perfectly competitive market. We add the government expenditure to the objective function; however, this does not mean that we treat the government expenditure as social welfare; instead, it reflects the distorted demand function.

### 3.3. Modeling Energy Crops and Conversion

Production activities for raising sweet potatoes, poplar and switchgrass as bioenergy feedstocks plus their conversion into ethanol and electricity are incorporated into the modified TASM. Here we discuss that modeling.

First, under current policy there is a substantial amount of set-aside land and these crops are modeled as using that land. Second in terms of crops sweet potatoes are currently produced in Taiwan, but not poplar and switchgrass. For this reason, input costs and yields for those crops are obtained from the literature and established models. Aylott *et al.* [39] showed that the yield of poplar is generally from 5.77 to 9.59 t/ha per year and this difference is caused by the quality of soil at the plantation and local weather. Sandy soil usually has the lowest yield. Since soil on Taiwan set-aside land is not sandy, this study takes average poplar yield. Switchgrass is a robust lowland energy crop most suited to the southern USA and has been tested in Auburn University test plots. In general, it has produces more than 10 tons per acre per year. Some U.S. government projects show that the yield of switchgrass is between 2 and 4 tons per acre per year and therefore, to be conservative, we use the average yield from government studies in our analysis. Since 1 hectare is 2.471 acres, the assumed annual yield of switchgrass is 3 × 2.471 = 7.4 t/ha per year. 

Third, in terms of transformation to energy ethanol and electricity transformations are included in the model (in US dollars). For sweet potato, we added a facility construction cost of NT$2.4 per liter and a processing cost of NT$8.4 per liter. We also added a hauling cost of NT$1 per liter of ethanol that was estimated following McCarl *et al.*’s 2000 [40] hauling cost formula. For poplar and switchgrass, ethanol cost data is from FASOM [41]. After adjusting for Taiwanese consumer price index, the processing cost (including fixed cost, hauling cost and other costs) is NT$12 per liter for poplar and NT$11 per liter for switchgrass. Since poplar and switchgrass are not planted in Taiwan, elasticities of demand are set equal to the elasticity of hardwood varieties. Outputs are calculated based on the data of Aylott *et al.* [39] while production costs are calculated based on the information from FASOMGHG [42]. Fertilizer and chemical costs per hectare are calculated to NT$11,885 for poplar and $18,763 for switchgrass. Per hectare energy and seed costs are calculated to NT$706 and NT$5,410 for poplar and NT$488 and NT$306 for switchgrass, respectively. We also compute the net mitigation of carbon dioxide using an estimate from Weber and Johannes [43]. They show that net carbon dioxide emissions are reduced by 0.107 ton per 1,000 liters of ethanol. Electricity generated from a kg of poplar is about 0.768 kWh and 0.919 kWh per kg for switchgrass. Therefore, a kg of poplar and switchgrass are equivalent to 0.125 kg and 0.149 kg of coal, respectively. McCarl [44] estimates that poplar can offset about 71.3% of carbon dioxide emissions relative to the fossil fuel, 83.4% and 75.1% for switchgrass and the associate emissions reduction is 0.28 kg CO_2_ per kg of poplar and 0.246 kg CO_2_ for switchgrass. 

### 3.4. Modeling Pyrolysis and Biochar

In this study, sweet potato, poplar and switchgrass are examined as potential pyrolysis feedstocks. Pyrolysis yields biooil, biogas and biochar. Following McCarl *et al.* [11] biooil and biogas are modeled as being used for bioelectricity generation, while biochar has multiple uses. First, biochar can be burned to provide electricity and reduce production cost. Second, biochar can be applied on cropland and obtain agricultural benefits such as higher crop yields and lower irrigation water use. 

Table 2 shows the pyrolysis outputs for sweet potato, poplar and switchgrass. The pyrolysis yields for sweet potato we used in this study are based on He *et al.* [45]. Pyrolysis yields of poplar are based on Bridgwater and Peacocke [46]. Because we analyze the different uses of biochar, the net electricity produced from pyrolysis will be different if biochar is not used for electricity generation. Data from Table 2 is further processed to remove water content from biooil, which better simulate the net electricity production. The lower heating value of the biochar, biooil and biogas are taken as 11.4 MJ per kg [11], 17.3 MJ per kg and 6.5 MJ per kg [47], respectively. By using these estimates, we calculate the electricity generated from the pyrolysis of each energy crop. 

**Table 2:** Outputs from Fast and Slow Pyrolysis.

Pyrolysis Type	Output	Poplar	Sweet Potato	Switchgrass
Fast Pyrolysis	Biooil	66%	87.56%	69%
	Biogas	13%	NA	11%
	Biochar	14%	12.44%	20%
Slow Pyrolysis	Biooil	56%	51.52%	58.55%
	Biogas	7%	34.99%	5.92%
	Biochar	31%	13.50%	44.29%

Source: [44,46].

### 3.5. Modeling GHG Emissions and Markets

The net GHG emissions offsets from pyrolysis, including both uses of biochar, are presented in Table 2. When burning biochar, all biochar is used to provide energy and therefore, we don’t need to consider hauling emissions. However, the GHG emissions offset from burning biochar in the pyrolysis plant as it displaces fossil fuels must be added. If biochar is used as a soil amendment, hauling is considered. Below presents the hauling cost for ethanol production. For sweet potatoes, we follow McCarl *et al.* [11] and assume that the ethanol plant is in the center of a square surrounded by a grid layout of roads. In turn, the hauling cost (H) and average hauling distance (*D*) is given by the following formula:

H = (b_0_ + 2b_1_*D*) S/Load (6)

and:

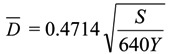
(7)
where *D* is the average distance that the feedstock is hauled in miles; S is the amount of feedstock input for a bio-refinery to fuel the plant, which we assume is 1 Mt (annual input) plus an adjustment for an assumed 5% loss in conveyance and storage; Load is the truck load size, which we assume to be 23 t (a general truck load size in Taiwan); Y is the crop yield (40 tons per ha per year) multiplied by an assumed crop (sweet potato) density of 38%; 640 is a conversion factor for the number of acres per square mile; B_0_ is a fixed loading charge per truckload and is assumed to be NT$2,700 per truckload for a 23 ton truck; and B_1_ is the charge for hauling including labor (per mile) and maintenance costs. Based on Chen’s estimation, we assumed it equal NT$66 and a 5% yield loss during transportation. 

Hauling cost of feedstock to pyrolysis plant follows the same methodology, given a needed feedstock production area of 1,268 ha of cropland this yields an average hauling distance of 2.7 km with a cost of NT$133.5 per ton. This cost stands for the hauling cost of transporting biomass to the pyrolysis plant. We also incorporate: (1) a cost of purchasing biochar and (2) a cost of hauling biochar from the plant to rice producing lands into the model. The biochar cost comes from its relationship with coal where it has about 40% of the energy content and with a coal price of NT$1 per kg we assume the biochar price is NT$1 per kg. Table 3 details the GHG offset potential of various feedstocks.

**Table 3:** Carbon Dioxide Offset from Burning Biooil, Biogas and Biochar (ton CO_2_ per ton of feedstock).

Type of Pyrolysis	Sweet Potato	Poplar	Switchgrass
**Pyrolysis optimized for energy**	0.31	0.4	0.418
**Pyrolysis optimized for biochar**	0.542	0.62	0.647

Source: Use Gaunt and Lehmann’s estimates [48] for GHG offset calculation.

## 4. Study Setup

This study examines Taiwan’s bioenergy production under alternative energy prices, and carbon prices. In particular we use three ethanol prices (NT$20, 30 and 40 per liter), two coal prices (NT$1.7 and 3.45 per kg), six GHG prices (NT$5, 15 and 30 per ton CO_2_e) and assumed GHG emissions from land use change. We also consider cases where the biochar is applied to land and where it is used to generate electricity. The study examines Taiwan’nudy examines Taiwanwd where it is used to generate electricity.ricity.t is used to generaticity, and GHG emissions offset by utilizing current set-aside land with the consideration of the emissions from fertilizer use and land use change. Three gasoline prices (NT$20, 30, 40 per liter), two coal prices (NT$1.7, 3.45 per kg), six GHG prices (NT$5, 10, 15, 20, 25, 30 per ton) plus estimated emissions from fertilizer use and land use change. The simulated gasoline and coal prices are selected based on the ranges of their market prices in 2012. Since Taiwan has not established a GHG trading mechanism and GHG emission is currently of no value in Taiwan, the study examines several potential GHG prices based on the opinion of Chen, who is familiar with and engaged in Taiwanese agricultural and environmental policies. 

GHG emissions from land use change are estimated by Liu *et al.* [3], who calculate that annual mean GHG fluxes from soil of plantation and orchard are 4.70 and 14.72 Mg CO_2_-C ha^−1^·yr^−1^, −2.57 and −2.61 kg CH_4_-C ha^−1^·yr^−1^ and 3.03 and 8.64 kg N_2_O-N ha^−1^·yr^−1^, respectively. Qin *et al.* [49] also indicated that the average N_2_O flux is 1.8 kg N ha−1 and most of the simulation results are less than 5 kg·N·ha^−1^. Because CO_2_ and N_2_O emissions are highly correlated with each other [25], we assume that the emission profile of CO_2_ and N_2_O are staying at the same level. In addition, Snyder *et al.* [50] show that fertilizer induced N_2_O emissions from soil equates to a GWP of 4.65 kg CO_2_ kg^−1^ of N applied. With these estimates, we arrive at the estimated emission level from fertilizer use and land use change (Table 4). 

**Table 4:** Net CO_2_e emissions from land use change under different GHG emission rates.

GHG	CO_2_	N_2_O	CH_4_	Net CO_2_e Emissions from Land Use Change
**Unit**	Mg ha^−1^·yr^−1^	kg ha^−1^·yr−1	kg ha^−1^·yr^−1^	Mg ha^−1^·yr^−1^
**Land GHG Emissions**	4.7	26.86	-2.57	11.62

The data on agricultural commodity market conditions largely are updates of that in TASM which was based on published government statistics and research reports including the FASOMGHG, Taiwan Agricultural Yearbook, Production Cost and Income of Farm Products Statistics, Commodity Price Statistics Monthly, Taiwan Agricultural Prices and Costs Monthly, Taiwan Area Agricultural Products Wholesale Market Yearbook, Trade Statistics of the Inspectorate-General of Customs, Forestry Statistics of Taiwan. 

## 5. Results

**Figure 1:**
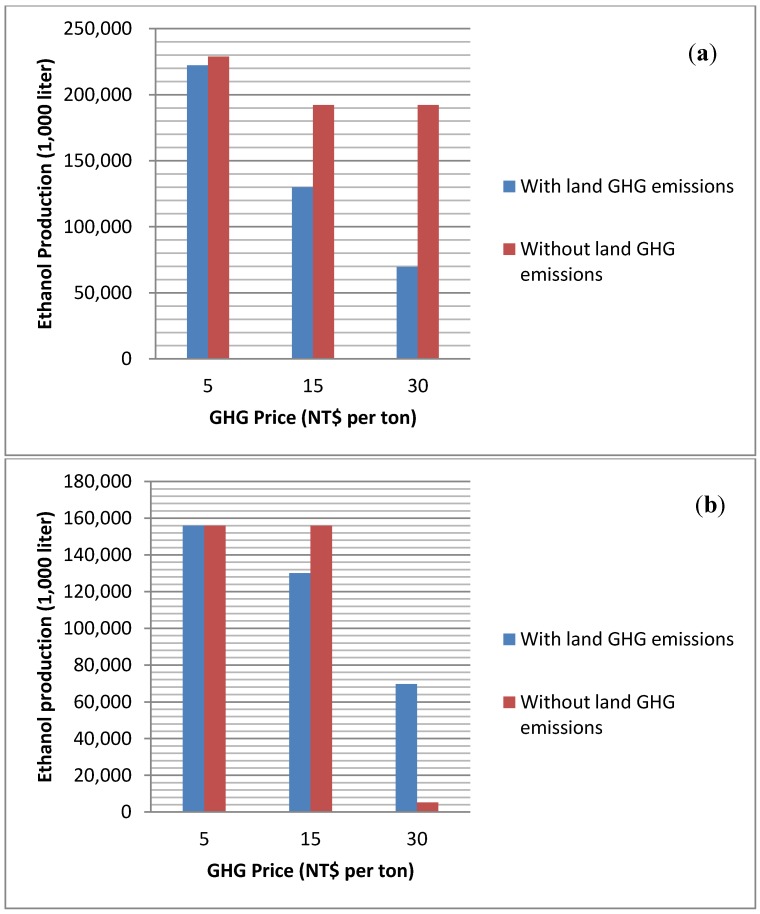
(**a**) Ethanol production when biochar is burned for energy; (**b**) Ethanol production when biochar is used as a soil amendment.

The simulation results indicate that only sweet potato should be used as a feedstock due to its higher production rate, lower cost per ton and harvest frequency (Appendix Table A1). Comparisons between bioenergy production and GHG emission reduction are also provided. Figure 1a,b shows the levels of ethanol production with and without endogenously incorporating land GHG emissions under various GHG prices. We find that when GHG prices increase, ethanol production decreases because ethanol offsets relative less GHG emissions than electricity and under higher GHG prices, ethanol production is replaced by pyrolysis-based electricity. Moreover, when biochar is used as an energy source, ethanol production is higher than when it is used as a soil amendment. This is explained by a combination of high returns to biochar use as a soil amendment, coupled with feedstock competition where more sweet potatoes are used for pyrolysis. However, if GHG price is low, ethanol is the better alternative. However, incorporation of land GHG emissions changes the ethanol production significantly. Ethanol production shrinks dramatically when land emissions are considered, especially for the burning biochar scenarios. This is because emissions from land-use change further reduces the net emissions offset of ethanol production and therefore, ethanol production drops to an even lower level at high GHG prices. 

**Figure 2:**
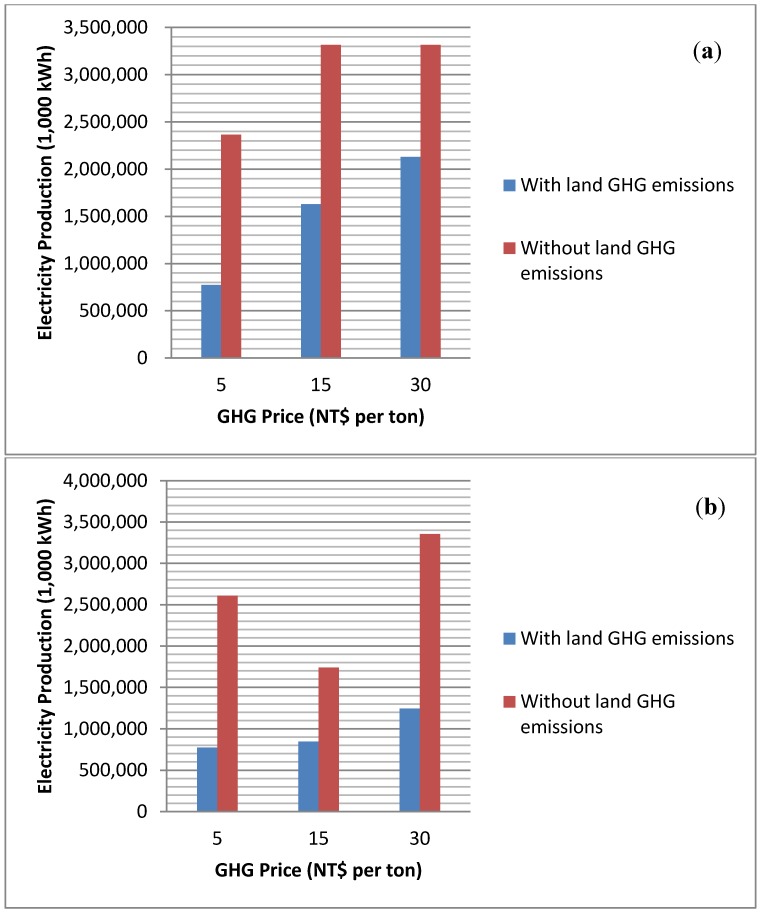
(**a**) Electricity production when biochar is burned for energy; (**b**) Electricity production when biochar is used as a soil amendment.

Figure 2a,b shows the levels of electricity production under alternative biochar uses. Here we see that when the GHG price is high, electricity production generally increases. When GHG price is low, producers will adopt fast pyrolysis and generate more electricity. However, when the GHG price becomes higher there is a switch to slow pyrolysis to yield more biochar with an associated reduction in electricity production but an increase in GHG emission offsets. This situation is more obvious when we compare the electricity production under land emissions scenarios. If land emissions are endogenously incorporated, electricity production is generally lower than that under no land emission scenarios. This is due to the higher GHG price has a significant impact on electricity production and hence more sweet potatoes are used in slow pyrolysis to gain better returns. This causes the net electricity production to decrease. 

**Figure 3:**
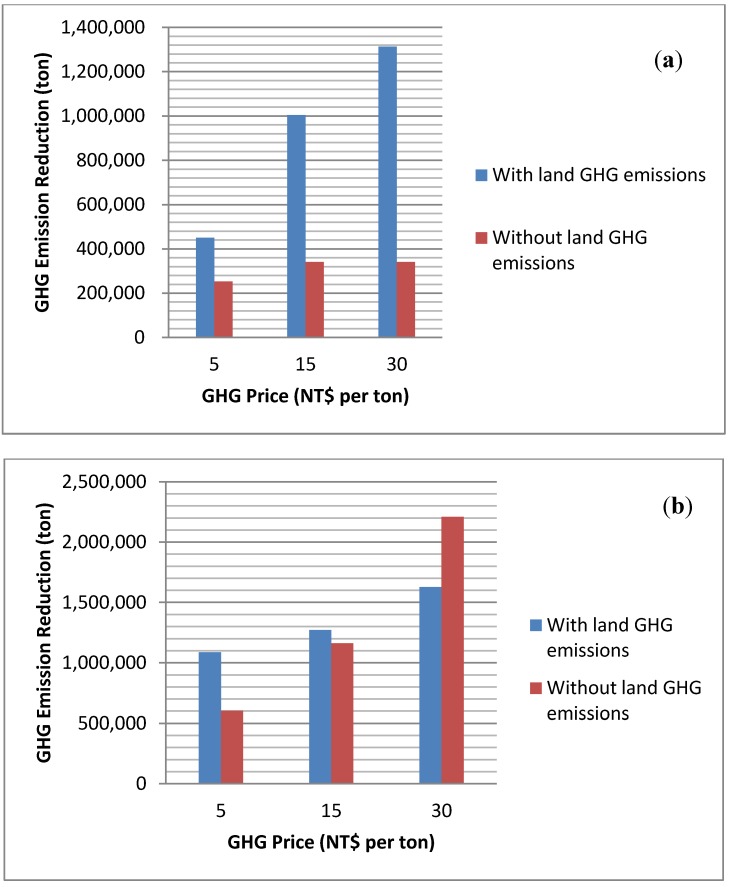
(**a**) Net GHG emissions offset when biochar is burned for energy; (**b**) Net GHG emissions offset when biochar is used as a soil amendment.

Figure 3a,b indicates the net GHG emissions from bioenergy production. As expected, net GHG emissions offsets increase when the GHG price increases. When land emissions are incorporated and biochar is applied to cropland, the highest net GHG emissions reductions are achieved. This result indicates that there is competition between domestic energy production and climate change mitigation. The result indicates that the largest net GHG emissions reduction occurs when Taiwan’s bioenergy production does not achieve the maximal. Slow pyrolysis and biochar application to crops is the best GHG alternative while fast pyrolysis with the biochar burned for electricity is the best energy alternative. The net GHG emission reduction of burning biochar scenarios when considering land emissions is higher than that in no land emissions scenarios because higher portion of electricity are produced from slow pyrolysis, which yield low electricity but high biochar. However, although slow pyrolysis produces more biochar, land emissions do offset the environmental benefits from using biochar as a soil amendment. Therefore, net emissions offset is lower when land emissions are incorporated.

The study also shows that when multiple bioenergy techniques such as ethanol, conventional electricity and pyrolysis-based electricity are competing with each other, conventional electricity is not competitive. The results also indicate that when land GHG emissions are incorporated, ethanol production decreases across all scenarios. Throughout all scenarios, simulation result can be interpreted as:

(1) When land GHG emissions are endogenously considered, electricity and ethanol production will have a significant shrink, compared to the no land emissions scenarios.
(2) When biochar is used as an energy source, more sweet potatoes are used in fast pyrolysis and lead to a larger reduction on ethanol production.
(3) Slow pyrolysis dominates fast pyrolysis, ethanol and conventional electricity at high GHG price. Fast pyrolysis dominates all other alternatives at low GHG price.
(4) Net GHG emission offset will increase in burning biochar scenarios but decrease in hauling biochar scenarios. The maximum amount of GHG emissions reduction from Taiwanese bioenergy production is lower when land emissions are endogenously considered into the production process.
(5) If land GHG emissions are not considered, simulation result for ethanol and electricity production and GHG emissions reduction will be too optimistic.

## 6. Conclusions

This study examined the trade-off relationship between Taiwanese bioenergy production and associated GHG emissions when ethanol, conventional bioelectricity and pyrolysis-based bioelectricity can be produced. The results show that when biochar is for electricity that, pyrolysis can provide up to 3.8 billion kWh or about 1.73% of Taiwan’s annual electricity demand while offsetting up to 2.2 million tons of GHG emissions (or 1.58% of Taiwan’s annual GHG emissions). 

We find that ethanol will not be competitive when land based emissions are considered under high GHG prices because of its low GHG offset levels. However, when ethanol price is higher than NT$40 per liter and GHG price is lower than NT$15 per ton, ethanol production is desirable and provides a substantial amount of ethanol for blended gasoline. Based on the simulation results, there are tradeoffs between energy production and GHG offsets and between electricity and ethanol production. 

Those crafting Taiwan’s bioenergy strategy may well want to consider these tradeoffs. Larger amounts of imported energy can be replaced with production of ethanol and fast pyrolysis based electricity but larger GHG emissions reductions occur with slow pyrolysis based electricity and land application of biochar. The result indicates that when land use emissions are endogenously incorporated, the best technique for climate change mitigation is slow pyrolysis and both ethanol and pyrolysis based electricity will decrease significantly. However, fast pyrolysis would still be a better technique for the concern of energy security and most of ethanol and conventional bioelectricity would be driven out. 

This study has limitations. The findings are a reflection of the assumptions used such as feedstock yields, energy recovery rates, pyrolysis yields and land emission rates. The accuracy of such assumptions could be improved. Also we also ignore the differences of weather and soil between counties that may actually lead to different crop yields and biochar application effects. These limitations can be addressed once related experimental data is available. 

## Glossary of Abbreviations

GHG: Greenhouse Gas
C: Carbon
N: Nitrogen
CO_2_: Carbon dioxide
CH_4_: Methane
N_2_O: Nitrous oxide

## Appendix

**Table A1:** Simulation results of bioenergy production and GHG emissions offset for with and without endogenous land GHG emissions.

When Biochar is Burned for Electricity		Unit					
	Ethanol Price	NT$ per liter	20	20	20	30	30
	Coal Price	NT$ per kg	1.7	1.7	1.7	1.7	1.7
	GHG Price	NT$ per ton	5	15	30	5	15
	Ethanol production	1,000 liter	222,347	130,000	69,550	236,740	156,000
With Land GHG	Electricity	1,000 kWh	773,500	1,627,877	2,130,621	773,500	1,430,823
	GHG emission reduction	ton	450,882	1,004,075	1,313,363	450,997	882,866
	Ethanol production	1,000 liter	228,822	192,163	192,096	306,243	269,840
Without Land GHG	Electricity	1,000 kWh	2,364,700	3,315,000	3,315,000	825,600	1,712,750
	GHG emission reduction	ton	253,214	340,569	340,562	108,743	195,062
	Coal Price	NT$ per kg	3.45	3.45	3.45	3.45	3.45
**When Biochar is Burned for Electricity**		**Unit**					
	GHG Price	NT$ per ton	5	15	30	5	15
	Ethanol production	1,000 liter	156,000	130,000	69,550	156,000	156,000
With Land GHG	Electricity	1,000 kWh	1,417,259	1,637,981	2,130,662	1,408,645	1,436,500
	GHG emission reduction	ton	824,125	1,010,301	1,313,389	819,125	886,364
	Ethanol production	1,000 liter	224,534	201,321	192,097	242,777	220,524
Without Land GHG	Electricity	1,000 kWh	2,475,200	3,094,000	3,315,000	2,408,900	2,983,500
	GHG emission reduction	ton	263,370	320,325	340,562	259,032	311,840
	Ethanol Price	NT$ per liter	30	40	40	40	
	Coal Price	NT$ per kg	1.7	1.7	1.7	1.7	
	GHG Price	NT$ per ton	30	5	15	30	
	Ethanol production	1,000 liter	130,000	238,218	240,546	130,000	
With Land GHG	Electricity	1,000 kWh	1,651,857	773,500	773,500	1,651,857	
	GHG emission reduction	ton	1,018,851	451,009	478,525	1,018,851	
	Ethanol production	1,000 liter	207,660	306,797	306,954	264,866	
Without Land GHG	Electricity	1,000 kWh	3,315,000	773,500	773,500	1,856,400	
	GHG emission reduction	ton	342,305	108,806	108,823	208,331	
**When Biochar is Burned for Electricity**		**Unit**					
	Ethanol Price	NT$ per liter	30	40	40	40	
	Coal Price	NT$ per kg	3.45	3.45	3.45	3.45	
	GHG Price	NT$ per ton	30	5	15	30	
	Ethanol production	1,000 liter	130,000	238,236	156,000	130,000	
With Land GHG	Electricity	1,000 kWh	1,651,898	773,500	1,435,372	1,651,897	
**When Biochar is Burned for Electricity**		**Unit**					
	GHG emission reduction	ton	1,018,876	451,009	885,669	1,018,876	
	Ethanol production	1,000 liter	207,660	251,907	235,769	208,261	
Without Land GHG	Electricity	1,000 kWh	3,315,000	2,187,900	2,607,800	3,315,000	
	GHG emission reduction	ton	342,305	238,785	277,390	342,372	
**When Biochar. is Hauled to Cropland**		**Unit**					
	Ethanol Price	NT$ per liter	20	20	20	30	30
	Coal Price	NT$ per kg	1.7	1.7	1.7	1.7	1.7
	GHG Price	NT$ per ton	5	15	30	5	15
	Ethanol production	1,000 liter	156,000	130,000	69,550	220,800	148,602
With Land GHG	Electricity	1,000 kWh	773,500	845,476	1,246,679	773,500	773,500
	GHG emission reduction	ton	1,088,257	1,271,000	1,627,488	552,498	1,232,109
	Ethanol production	1,000 liter	156,000	156,000	5,200	284,650	156,000
Without Land GHG	Electricity	1,000 kWh	2,607,800	1,740,322	3,353,446	773,500	1,743,157
	GHG emission reduction	ton	603,841	1,163,300	2,208,492	216,557	1,165,167
**When Biochar. is Hauled to Cropland**		**Unit**					
	Ethanol Price	NT$ per liter	20	20	20	30	30
	Coal Price	NT$ per kg	3.45	3.45	3.45	3.45	3.45
	GHG Price	NT$ per ton	5	15	30	5	15
	Ethanol production	1,000 liter	156,000	69,550	69,550	156,000	130,000
With Land GHG	Electricity	1,000 kWh	773,500	1,314,196	1,288,531	773,500	844,497
	GHG emission reduction	ton	1,048,141	1,550,040	1,642,042	1,098,684	1,269,077
	Ethanol production	1,000 liter	179,070	156,000	5,200	202,828	156,000
Without Land GHG	Electricity	1,000 kWh	2,519,400	1,740,847	3,308,116	2,475,200	1,745,286
	GHG emission reduction	ton	587,067	1,163,646	2,178,646	564,761	1,166,568
**When Biochar. is Hauled to Cropland**		**Unit**					
	Ethanol Price	NT$ per liter	30	40	40	40	
	Coal Price	NT$ per kg	1.7	1.7	1.7	1.7	
	GHG Price	NT$ per ton	30	5	15	30	
	Ethanol production	1,000 liter	130,000	275,667	156,000	156,000	
With Land GHG	Electricity	1,000 kWh	808,214	151,032	773,500	773,500	
	GHG emission reduction	ton	1,333,213	350,500	1,093,777	1,227,404	
	Ethanol production	1,000 liter	130,000	287,430	156,000	156,000	
Without Land GHG	Electricity	1,000 kWh	2,043,067	773,500	1,748,897	1,788,248	
	GHG emission reduction	ton	1,359,715	201,581	1,168,946	1,194,854	
**When Biochar. is Hauled to Cropland**		**Unit**					
	Ethanol Price	NT$ per liter	30	40	40	40	
	Coal Price	NT$ per kg	3.45	3.45	3.45	3.45	
	GHG Price	NT$ per ton	30	5	15	30	
	Ethanol production	1,000 liter	130,000	220,819	156,000	130,000	
With Land GHG	Electricity	1,000 kWh	819,030	773,500	773,500	819,030	
	GHG emission reduction	ton	1,361,671	552,499	1,105,395	1,361,671	
	Ethanol production	1,000 liter	130,000	217,112	156,000	156,000	
Without Land GHG	Electricity	1,000 kWh	2,028,091	2,165,800	3,274,577	1,788,649	
	GHG emission reduction	ton	1,349,855	498,605	776,957	1,195,118	
